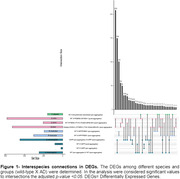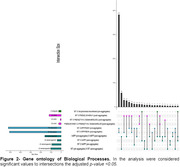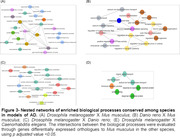# Differentially expressed genes and biological processes shared by alternative and mouse models of Alzheimer’s disease

**DOI:** 10.1002/alz.092626

**Published:** 2025-01-03

**Authors:** Flávia Suelen De Oliveira Pereira, Giovanna Carello‐Collar, Vitória Vitalina Margarita Ryssina, Daiana Silva Ávila, Marco Antônio de Bastiani, Eduardo R. Zimmer

**Affiliations:** ^1^ Universidade Federal do Pampa, Uruguaiana, Rio Grande do Sul Brazil; ^2^ Universidade Federal do Rio Grande do Sul, Porto Alegre, RS Brazil; ^3^ Universidade Federal de Ciências da Saúde de Porto Alegre, Porto Alegre, Rio Grande do Sul Brazil; ^4^ Universidade Federal do Rio Grande do Sul, Porto Alegre, Rio Grande do Sul Brazil; ^5^ Universidade Federal do Rio Grande do Sul, Porto Alegre Brazil; ^6^ Brain Institute of Rio Grande Do Sul, PUCRS, Porto Alegre, RS Brazil; ^7^ McGill Centre for Studies in Aging, Montreal, QC Canada

## Abstract

**Background:**

Replacement, Reduction, and Refinement (3R) guidelines propose the use of alternative models to study human diseases. These models have high homology and are less onerous compared to rodents, which dominate Alzheimer’s disease (AD) research. However, it is still necessary to investigate whether evolutionary components are conserved among AD models cross‐species. Thus, we aimed to determine similar and different core molecular programs and biological processes in alternative and mouse models of AD.

**Methods:**

We searched the Gene Expression Omnibus (GEO) repository for available RNa‐sequencing studies of alternative (*Caenorhabditis elegans*, *Drosophila melanogaster*, and *Danio rerio*) and mouse (*Mus musculus*) models of amyloidosis. The following datasets were selected for alternative (GSE198684, GSE109489, GSE158233) and mouse (GSE186710 and GSE144746) models of AD. According to the species, we selected the brain (hippocampus), head, or whole body as samples. Data was downloaded using the GEOquery package and the differentially expressed genes were defined as having FDR‐ adjusted *p‐value* < 0.05. Using the R Studio and Cytoscape software, we grouped DEGs into clusters of gene ontology for biological processes (GOBPs) according to their function.

**Results:**

A greater number of DEGs were shared between *D. rerio* and mouse models of amyloidosis when compared to other species (Figure 1). By comparing alternative models, *C. elegans* has more DEG‐intersections with *D. melanogaster than with D. rerio* (Figure 1). Regarding biological processes, more overlap of GOBPs was found between *D. melanogaster and M. musculus* (Figure 2). *C. elegans*, despite having present DEGs in common with *D. rerio and M. musculus*, did not share GOBP terms with these vertebrates, but only with *D. melanogaster* (Figure 2). The shared biological processes conserved among the species were classically associated with AD pathology, such as protein misfolding response, immune and inflammatory systems, neurotransmission, and metabolic processes (Figure 3).

**Conclusions:**

The findings suggest that alternative models share transcriptomic similarities with mouse models of amyloidosis. Thus, these models may serve to accelerate the understanding of amyloid‐related pathophysiological processes and the development of innovative therapeutics in AD.